# Transactions of the Chicago Society of Physicians and Surgeons, February 9th, 1874

**Published:** 1874-03

**Authors:** Plym. S. Hayes


### Society Reports.

### TRANSACTIONS OF THE CHICAGO SOCIETY OF PHYSICIANS AND SURGEONS.

### MEETING OF FEBRUARY 9TH, 1874.

*Reported by Plym. S. Hayes, M.D.*

THE Society met, as ususal, in the parlor of the Grand Pacific Hotel, the President, Dr. A. Fisher, in the chair.

Drs. J. H. Hollister, and W. T. Montgomery, were unanimously elected to membership.

Dr. Hyde read the following communication :

CHICAGO, ILL., Feb. 9, '74.

*Dear Sir* :—I have the pleasure of presenting to the Chicago Society of Physicians and Surgeons, in behalf of Assistant Surgeon John S. Billings, U. S. A., the Librarian, the accompanying two volumes of the catalogue of the Library of the Surgeon General's Office, U. S. Army.

Very Respectfully,

W. C. SPENCER,

*Surg. U. S. A.*

DR. J. N. HYDE,

*Sec'y Chi. Soc'y Phys. and Surg.*

A vote of thanks was tendered to the Surgeon General

Dr. Danforth's report on the Pathology of Endemic Cholera was read by Dr. Bridge. The report mainly consisted of the history, necroscopy, and microscopical examination of two cases of patients that died in the cholera hospital last summer.

Dr. Danforth explained the sections of normal and pathological intestines, which he had prepared. These were projected on a screen, by means of a solar microscope. The instrument used was one of Browning's spectroscopic lanterns, with microscopic attachment. The lantern had been recently presented to Rush Medical College by Mr. A. C. Thomas, and kindly loaned to the Society by the College.

A vote of thanks was given to Dr. Danforth for the paper and exhibition of microscopic illustrations.

The following resolution was adopted:

*Resolved*, That the report be given to the Secretary for publication in some medical journal.

As the hour was late, the discussion of the paper was made the business of the next meeting.

The meeting then adjourned.

